# Phenolic content, chemical composition and anti-/pro-oxidant activity of Gold Milenium and Papierowka apple peel extracts

**DOI:** 10.1038/s41598-020-71351-w

**Published:** 2020-09-11

**Authors:** Monika Kalinowska, Kamila Gryko, Anna M. Wróblewska, Agata Jabłońska-Trypuć, Danuta Karpowicz

**Affiliations:** 1 Department of Chemistry, Biology and Biotechnology, Bialystok University of Technology, Wiejska 45A, 15-351 Bialystok, Poland; 2 Faculty of Chemistry, Warsaw University of Technology, Noakowskiego 3, 00-664 Warsaw, Poland

**Keywords:** Preventive medicine, Secondary metabolism, Chemical biology, Natural products

## Abstract

In this study the peels of ecologically grown apple (*Malus domestica*) cultivars: Gold Milenium (a new scab‐resistant variety) and Papierowka (Papirovka; an old, sensitive to apple scab variety) were examined for their composition (phenolic compounds, triterpenoids, simple organic acids, macro-, microelements, reducing sugars, l-ascorbic acid), pro- and antioxidant properties as well as their application in reduction of the oxidative stress in cultured human skin fibroblast. The higher content of phenolic compounds correlated with the greater pro- and antioxidant activity of the peels of Papierowka compared to Gold Milenium in DPPH·, ABTS^+^, FRAP and CUPRAC assays as well as an ability to inhibition of lipid peroxidation. The quantity of the compounds strongly depended on the type of extraction. The extract of Papierowka peels possessed much higher amount of phenolic compounds compared to Gold Milenium (Papierowka: 3.68 ± 0.20 mg/g peel ultrasound assisted extraction (u.a.e); 2.02 ± 0.13 mg/g peel conventional extraction (c.e.); Gold Milenium: 1.46 ± 0.19 mg/g peel u.a.e; 1.15 ± 0.04 mg/g peel c.e. according the HPLC measurement). The pro-oxidant activity of the extract from Papierowka peels can be correlated with the content of phenolic compounds and metal ions as well. The apple peel extract is promising agent reducing the oxidative stress in skin fibroblast.

## Introduction

Plant phenolic compounds provide protection against environmental stress factors. They are synthesized in response to infectious diseases caused by pathogens (e.g. bacteria, fungi, viruses, worms), UV radiation, climate conditions or toxic metals^1,2^. The mechanism of the protective action of phenolic compounds in plants rely on: (i) antioxidant activity that scavenges free radicals, protection of lipid peroxidation, (ii) chelation of toxic metals, (iii) participation in lignification process^2^. The varieties of plants with enhance content of phenolic compounds are of great interest because they adapt more easily to environmental factors in organic farming. Moreover, food rich in phenolic compounds are desired by consumers for maintaining a good health and preventing diseases induced by free radicals, including cancer, artherosclerosis, diabetes mellitus, Alzheimer. Plant extract and individual phenolic compounds can be applied as antioxidants, preservatives or drugs, e.g. plant phenolic compounds can protect skin exposed to the polluted atmospheric environment and skin diseases^3^.

**Figure 1:**
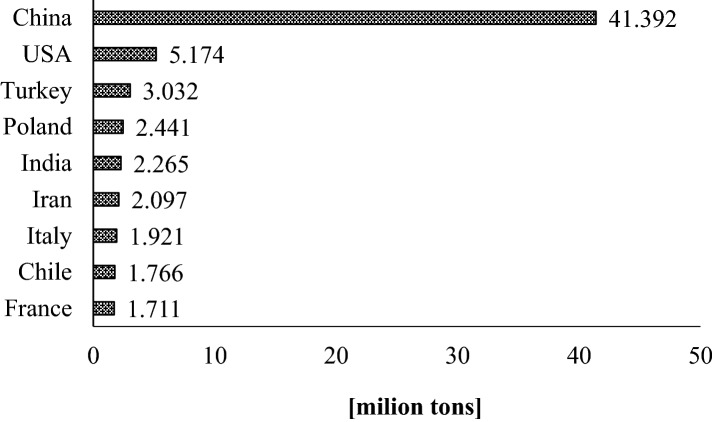
The worldwide production of apples in the 2017 year according to Food and Agriculture Organization of the United Nation (FAO)^14^.

According to McCann’a et al. apples contain more free phenolic compounds than other popular fruits^4^. Moreover, an apple is a source of fiber, macro- and micronutrients, sugars and phenolic compounds^5^. Several studies reveal that consumption of apples decreased risk of lung cancer^6^, colon and colorectal cancers^7^, prostate cancer^8^, breast cancer^9^. Another study shown that apple consumption is linked with a decrease risk of asthma^10^. Apples lower the level of cholesterol and triglycerides in the blood serum and reduced risk of cardiovascular diseases as well as help in lost weight and reduced risk of diabetes^11,12^. What is important, the pro-healthy properties of apples depend on their chemical composition, which in turn strongly depends on the variety, weather conditions, maturity of fruit, illnesses, fruit cultivation system, mode and time of storage and processing^13^. Poland is the biggest apple producer in the European Union, ahead of Italy and France^14^. Taking into account the global production of apples, Poland is the fourth in the world producer of these fruits (Fig. 1). The most popular apple varieties and most often bought by consumers are Golden Delicious, Red Delicious and Gala. They are chosen because of the great taste and attractive appearance, but there are not characterized by the highest content of pro-healthy compounds (phenolic compounds) among other apples. Many new apple varieties rich in antioxidants and resistant to illness were selected so far. Nevertheless the old apple varieties are intensively studied because they possess the natural resistance to environmental stress factors. Moreover, many studies revealed that old varieties of apple possess higher content of phenolic compounds than the new ones^12,15^.

Selected in this study Gold Milenium is a scab resistant, relatively new variety selected in Research Institute of Horticulture in Skierniewice (Poland). It is a late summer variety, not available for purchase in common supermarkets. Papierowka (Papirovka) is an old, highly resistant to frost and sensitive to apple scab variety. Our preliminary studies showed that the content of antioxidant is much more higher in peel and core than flesh of apples, therefore the apple peels were intensively studied in this paper. Moreover the type of extraction strongly affects the release of antioxidants. The application of ultrasounds enhances the diffusion of solvent through the cell walls and the releasing of the cell components^16^. The process of ultrasound extraction of polyphenols from different plant sources was optimized by many authors^17–20^.

In this paper two apple varieties were compared due to chemical composition and antioxidant activities. Moreover the pro-oxidant action of the extract (which depends on e.g. the concentration or the presence of transition metals) was also examined. The effect of the type of extraction (conventional vs. ultrasound assisted) on the composition of extracts and their biological activities was evaluated. The potential application of Gold Milenium and Papierowka apple peels as a source of antioxidant, skin fibroblast protectors in protection against negative effects of the environment was discussed.

## Materials and methods

### Plant material

Gold Milenium and Papierowka apples were picked at maturity, in 2017, from organic farm located in Podlasie province. Five to seven kg of apples were harvested. The apples were washed, dried and peeled. The fresh peels were dried in oven with ventilation system (Venticell 55) at 36 °C for 24 h. The moisture content of peels was 14% (measured with the use of MA 50/1.R Radwag moisture analyser). The dried peels were ground using a laboratory grinder IKA A 11 analytical mill.

### Chemicals

All chemicals have an analytical purity and were used without further purification. Iron(III) chloride (FeCl_3_·6H_2_O), iron(III) sulfate (FeSO_4_), trolox, H_2_O_2_, horse radish peroxide, phosphate buffer pH 7, Folin-Ciocalteu reagent, DPPH (2,2-diphenyl-1-picrylhydrazyl), 2,4,6-tripyridyl-*s*-triazine (TPTZ), trichloroacetic acid (TCA), thiobarbituric acid (TBA), sodium dodecyl sulphate (DSD) and dichlorodihydrofluorescein diacetate assay (DCFH-DA) were purchased from Sigma-Aldrich Co. (St. Louis, MO, USA). Ethanol and methanol were bought from Merck (Darmstadt, Germany). Dulbecco’s modified Eagle’s medium (DMEM) containing glucose at 4.5 mg/mL (25 mM), penicillin, streptomycin, trypsin–EDTA, fetal bovine serum (FBS) and phosphate buffered saline (PBS) without Ca and Mg were purchased from Gibco (San Diego, USA). DTNB (dithiobis-2-nitrobenzoic acid, Ellman's reagent) was provided by Serva Electrophoresis GmbH (Heidelberg, Germany). The fibroblasts cell line was obtained from American Type Culture Collection (ATCC).

### Macro-, microelements and sugar determination

To determine the content of copper, manganese and iron, 5 g of air-dried sample was weighted in porcelain crucible and subjected to mineralization at 450 °C. After cooling down to room temperature, 5 mL of HCl (1:1) was added and the solution was transferred into the 50 mL volumetric flask. The measurement was done using atomic absorption spectrometer Varian SpectrAA 880 at 324.8 nm (for copper), 279.5 nm (for manganese) and 248.3 nm (for iron determination). To determine to content of calcium, magnesium, sodium and potassium, the 0.5 g of air-dried sample was mineralized with H_2_SO_4_ and 30% H_2_O_2_ and then diluted to 250 mL. The quantity of calcium, magnesium, sodium and potassium was determined with the use of atomic absorption spectrometer Varian SpectrAA 880 at 422.6 nm, 285.2 nm, 589.6 nm and 766.5 nm, respectively. The content of saccharose and reducing sugar was determined according to Lane–Eynon method^21^.

### Methods of extraction

An accurately weighed 2 g of sample (with the accuracy of 0.001 g) were extracted three times using each time 50 mL portions of solvent. The condition of extraction was optimized taking into account: solvent (water, methanol, ethanol, acetone, diethyl ether), time of extraction (30 min–4 h) and temperature (20–100 °C depending on the type of solvent). The efficiency of extraction was measured as the total content of phenolic compounds according to the methodology with Folin–Ciocalteu reagent^22^. The higher value of the total content of phenolics the more effective is the extraction process. The best parameters for apple extraction were: 80% methanol or 80% ethanol (ethanol was chosen as less toxic solvent), 1.5 h of total extraction (3 × 30 min) at the maximum temperature (60 °C was chosen for ethanol). The extraction was conducted in amber borosilicate glass bottles with caps using ultrasound water bath POLSONIC SONIC-3 (ultrasound frequency 40 kHz). Then the solvent was evaporated using Heidolph Hei-VAP Advantage ML/HB/G3 rotary evaporator. To obtain dry extract the residue was kept in oven with ventilation system at 36 °C for 24 h. Then the samples were stored in a refrigerator (3–4 °C) and the appropriate solutions in 80% ethanol were made before the analysis. The process of extraction was made in triplicate for each apple variety.

### HPLC analysis

High*-*performance liquid chromatography with diode*-*array detection was used for the determination of phenolic compounds in apple peel extracts obtained by standard and ultrasound assisted extraction. The column Zorbax Eclipse Plus C18 Analytical column (4.6 × 250 mm; 5 μm). The injected volume of samples was 10 μL. The concentration of samples was 0.7–0.8 g of solid extract per 10 mL of methanol. For phenolic compounds analysis the mobile phase consisted of acetonitrile (A) and 2% acetic acid (B) at flow rate of 1 mL/min. The gradient program of elution was used: 0–40 min, linear gradient from 3 to 15% A and from 97 to 85% B; 40–60 min, linear gradient 15–25% A and 85–75% B; 60–75 min: linear gradient 25–50% A and 75–50% B; 75–80 min: linear gradient 50–95% A and 50–5% B; linear gradient 80–85 min: 95–3% A and 5–97% B; 85–90 min: 3% A and 97% B. The detector was set at 280, 320 and 360 nm. For triterpenoids the mobile phase was 90% acetonitrile and 10% water, isocratic elution, mobile phase speed: 0.5 mL/min, sample volume: 10 μL, analytical wavelength: 280 nm. For analysis of organic acids and ascorbic acid: the mobile phase 50 mM H_3_PO_4_ and 10 mM NaH_2_PO_4_, isocratic elution, mobile phase speed 0.8 mL/min, sample volume 10 μL, analytical wavelength 210 and 244 nm. The identification was done on the basis of retention time and DAD absorbance spectra of standards. The content of chemical compounds was done with the five point calibration curve of standards.

### Total content of antioxidants

Total content of antioxidants (TCA) was determined according to the method described by Singleton and Rosi^23^. The total content of phenolic compounds was expressed as gallic acid equivalent, i.e. mg GA/g of extract (the standard curve equation: y = 0.137x − 0.0036; R^2^ = 0.9997) or mg GA/g dry weight of sample (d.w.). The experiment was done in five repetitions for three independent samples for each apple variety.

### Anti-/pro-oxidant activity

The antiradical activity was performed according to the DPPH assay described previously^24^. The percentage inhibition of DPPH radicals (%I) was calculated according to the equation:

$$\mathrm{\% I}=\frac{{\mathrm{A}}_{\mathrm{control}}^{516}-{\mathrm{A}}_{\mathrm{sample}}^{516}}{{\mathrm{A}}_{\mathrm{control}}^{516}}\times 100\mathrm{\%}$$

where: ${\mathrm{A}}_{\mathrm{conrol}}^{516}$—absorbance of the control sample, ${\mathrm{A}}_{\mathrm{sample}}^{516}$—absorbance of the tested sample. The concentration of the extracts was plotted against the % inhibition and the EC_50_ values were determined by linear regression analysis.

The antiradical activity against ABTS radical was measured according to Re et al.^25^ and expressed as the percentage inhibition of ABTS radicals (%I):

$$\mathrm{\% I}=\frac{{\mathrm{A}}_{\mathrm{control}}^{734}-{\mathrm{A}}_{\mathrm{sample}}^{734}}{{\mathrm{A}}_{\mathrm{control}}^{734}}\times 100\mathrm{\%}$$

where: ${\mathrm{A}}_{\mathrm{conrol}}^{734}$—absorbance of the control sample, ${\mathrm{A}}_{\mathrm{sample}}^{734}$—absorbance of the tested sample. The EC_50_ parameter was determined as it was described above.

Ferric reducing antioxidant activity was determined in FRAP assay^24^. Antioxidant activity was expressed as Fe^2+^ equivalents [μM] using the calibration curve prepared over the range of 1,000—20 μM of FeSO_4_ (the standard curve equation: y = 3,296.9x − 0.0331; R^2^ = 0.9997).

Cupric reducing antioxidant activity (CUPRAC) assay was conducted according to^26^. Antioxidant activity was expressed as trolox equivalents [μM] by using the calibration curve obtained for trolox in the range of concentration 50–250 μM (the standard curve equation: y = 254.7c − 0.0249; R^2^ = 0.9979).

The ferric thiocyanate method was used^27^. The % inhibition of lipid peroxidation (%P) was calculated:

$$\mathrm{\% }P=\frac{{\mathrm{A}}_{\mathrm{control}}^{500}-{\mathrm{A}}_{\mathrm{sample}}^{500}}{{\mathrm{A}}_{\mathrm{control}}^{500}}\times 100\mathrm{\%}$$

where:${\mathrm{A}}_{\mathrm{conrol}}^{500}$—absorbance of the control sample, ${\mathrm{A}}_{\mathrm{sample}}^{500}$—absorbance of the tested sample.

The pro-oxidant activity of extracts were measured as the rate of trolox oxidation according to the procedure described previously^28^.

The measurements were conducted using the Agilent Carry 5000 spectrophotometer (CA, USA). All experiments were done in five repetitions for three independent samples for each apple variety.

### Cytotoxicity study

Cytotoxicity analysis was conducted by using MTT assay, according to/as described previously in^29^.

### Determination of SH groups and TBA reactive species (TBARS) levels

Fibroblast cells were maintained in DMEM containing 10% FBS, penicillin (100 U/mL), and streptomycin (100 μg/mL) at 37 °C in a humified atmosphere (5% CO_2_ in air). Extracts were prepared before analysis from solid residue in DMSO and stored in a refrigerator at temperature 4 °C. The extract was added to the cultured cells for a final concentration 100 and 200 μg/mL. The control cells were incubated without the test compounds. Fibroblasts (1 × 10^5^ cells/mL) were incubated in 2 mL of culture medium with tested extracts in tissue culture 6-well plates. The determination of protein concentration was performed according to the method described by Lowry et al. as described previously^29^. SH-groups and the level of membrane lipid-peroxidation products (TBARS) were measured using the method of Rice-Evans^29^. All the experiments were done in triplicates.

### Statistical analysis

The statistical analysis was done in Statistica 13.0. The analysis of variance ANOVA and Tukey’s test were applied. Results from three independent experiments were shown as mean ± standard deviation of mean. A value of p ≤ 0.05 was considered significant. Differences between treatments and untreated control cells in MMT assay were analyzed by a one-way ANOVA, followed by a Dunnett’s procedure for multiple comparisons. Significant effects are represented by p ≤ 0.05(*) and p ≤ 0.01 (**).

## Results

### Macro-, microelements and sugar content

The composition of apple depends on their variety and the part of an apple (Table 1). Peels were more abundant in macro-, microelements and sugars than flesh (only in the case of Papierowka the content of saccharose was higher in the peel than the flash). Papierowka peels contained more iron, copper, potassium and reducing sugars, but higher amount of manganese and saccharose was determined in Gold Milenium peels. The reverse situation was in the case of flesh—Gold Milenium had higher (or almost equal) content of iron, copper, manganese and potassium than Papierowka. Moreover the flesh of Gold Milenium apple was more abundant in saccharose and less in reducing sugars. Determination of mineral and sugar content in samples is very important due to the possible interaction of the macro-. Microelements and sugars with the reagents during the next analytical assignments.

**Table 1:** The content of iron, copper and manganese [mg/kg] as well as sodium, potassium magnesium, calcium and sugars [%] in peel and flesh of Papierowka and Gold Milenium.

Variety	Fe	Cu	Mn	Na	K	Mg	Ca	Saccharose	Reducing sugars
	[mg/kg]			%					
**Papierowka**									
Peel	16.1	3.54	1.18	< 0.01 (0.001)	0.37	< 0.05 (0.019)	< 0.04 (0.011)	3.1	5.5
Flesh	1.57	0.17	0.50	< 0.01 (0.0006)	0.14	< 0.05 (0.005)	< 0.04 (0.006)	2.3	7.9
**Gold Milenium**									
Peel	7.97	2.13	2.35	< 0.01 (0.002)	0.24	< 0.05 (0.028)	< 0.04 (0.025)	4.1	4.6
Flesh	2.42	0.20	0.49	< 0.01 (0.0005)	0.14	< 0.05 (0.007)	< 0.04 (0.006)	5.5	6.4

### Total content of antioxidants (TCA)

**Figure 2:**
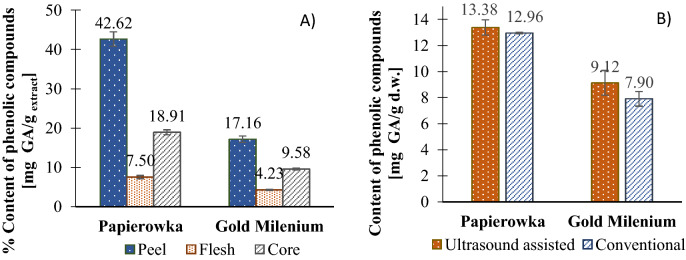
(**A**) The % content of phenolic compounds [mg GA/g d.w. peels] depending on the variety and the part of apple. (**B**) The content of phenolic compounds [mg GA/g d.w. peels] depending on the apple variety and the type of extraction.

The total content of antioxidants in Papierowka was higher than in Gold Milenium depending on the part of apple (Fig. 2A). Regardless the apple variety the TCA increased as follows: flesh < core < peel (Fig. 2A). The results showed that extracts obtained by ultrasound assisted extraction possess slightly higher content of antioxidants (Papierowka: 13.308 ± 0.584; Gold Milenium: 9.123 ± 0.944 mg GA/g d.w. peels) than the ones obtained by a conventional way (Papierowka: 12.957 ± 0.068; Gold Milenium: 7.902 ± 0.318 mg GA/g d.w. peels) (Fig. 2B).

The assay with the Folin–Ciocalteu (F–C) reagent is commonly applied to the study of the total content of phenolic compounds in food products although it is well known that the other non-phenolics react as well with the F–C reagent, i.e. some of the vitamins and their derivatives (l-ascorbic acid, folic acid, folinic acid, retinoic acid, thiamine), nucleotide bases (e.g. guanine), amino acids (tyrosine, tryptophan, cysteine) and simple inorganic ions (Fe^2+^, Mn^2+^, I^−^ and SO_3_^2−^)^30^. Moreover some metals may form complexes with phenolic compounds and change their redox potential. On the other hand linoleic, linolenic acids, fructose, glucose, sucrose, potassium nitrite, and organic acids has no reactivity toward F–C reagent^30^. Therefore is it obvious that the assay with the F–C reagent may overestimate the content of phenolic compounds. Because the phenolic compounds are the most abundant antioxidants in plant, it is assumed they have the largest contribution to the value obtained by assay with F–C reagent.

### HPLC analysis of phenolics, triperpenoids and organic acids

The HPLC analysis showed that the total content of phenolic compounds (TCPC) in the extract of Papierowka peels was 10.195 ± 0.567 mg/g dry weight (d.w.) of extract (ultrasound assisted extraction; u.a.e.) and 5.559 ± 0.366 mg/g d.w. of extract (conventional extraction; c.e.) depending on the type of extraction. In the case of Gold Milenium peels the value of TCPC = 3.934 ± 0.505 mg/g d.w. of extract (u.a.e.) and 3.001 ± 0.094 mg/g d.w. of extract (c.e.) (Table 2). The apple peel extracts were abundant in: chlorogenic acid, homovanilic acid, (−)epicatechin, rutin, quercetin-3-glucoside, quercetin-3-rhamnoside, phloridzin, ellagic acid. Although the extract of Papierowka peels possessed higher total content of phenolic compounds, some of the individual phenolics were more abundant the peel extract of Gold Milenium variety, especially ellagic acid and (−)epicatechin. The type of extraction strongly affected the efficiency of phenolic content in the peel extracts, especially in the case of Papierowka variety—the application of ultrasounds increased by 83% the content of phenolic compared to the conventional extraction (and 31% in the case of Gold Milenium peel extract) (Figs. S1, S2). What is interesting, some of the individual phenolic compounds were more abundant in the extracts obtained conventionally regardless of the apple variety. These were simple phenolic acids, i.e. gallic, protocatechuic and chlorogenic acids as well as quercetin in the case of Papierowka variety. The other phenolics listed in the Table 2 were more efficiently extracted from the apple peels by the use of ultrasounds. Especially in the case of Papierowka variety, the differences between the content of phenolic compounds in the extracts obtained by these two ways of extraction were very significant. In Table 3 the content of selected terpenoids and organic acids (including ascorbic acid) in apple peels is shown. Papierowka peels were more abundant in the determined compounds except oleanolic acid. The conventional extraction gives better results in the extraction of majority of these compounds (Table 3).

**Table 2:** The HPLC analysis of phenolic compounds in the of Papierowka and Gold Milenium apple peels [μg/g d.w. extract or peel].

TR [min]	λ [nm]	Phenolic compound	Papierowka					Gold Milenium			
			Ultrasound		Conventional			Ultrasound		Conventional	
			μg/g d.w. extract	μg/g d.w. peel	μg/g d.w. extract		μg/g d.w. peel	μg/g d.w. extract	μg/g d.w. peel	μg/g d.w. extract	μg/g d.w. peel
		**Phenolic acids**									
5–7	280	Gallic acid	3.23 ± 0.79	1.17 ± 0.28	8.18 ± 1.92		2.97 ± 0.70	4.47 ± 0.40	1.66 ± 0.15	4.88 ± 3.14	1.87 ± 1.20
9–11	280	Protocatechuic acid	53.89 ± 6.74	19.46 ± 2.43	57.67 ± 1.98		25.21 ± 7.42	6.12 ± 2.74	2.28 ± 1.02	41.14 ± 5.522	15.80 ± 2.12
20–22	320	Chlorogenic acid (5-caffeoylquinic acid)	467.80 ± 67.82	168.95 ± 24.50	930.14 ± 11.62		337.64 ± 3.07	473.91 ± 49.37	176.41 ± 18.38	450.59 ± 11.12	173.03 ± 4.27
22–24	280	Vanilic acid	150.31 ± 28.83	54.29 ± 10.41	21.37 ± 1.59		10.89 ± 5.45	23.15 ± 3.60	8.62 ± 1.34	21.13 ± 9.91	8.11 ± 3.81
25–27	280	Homovanilic acid	747.06 ± 59.50	269.81 ± 21.49	382.98 ± 14.88		139.02 ± 5.40	446.08 ± 35.76	166.05 ± 13.31	174.31 ± 33.92	66.93 ± 13.03
44–45	280	Ellagic acid	81.99 ± 3.96	29.6149 ± 1.43	75.17 ± 0.60		19.73 ± 13.08	464.75 ± 70.95	173.00 ± 26.41	416.97 ± 19.99	160.12 ± 7.68
57–58	320	Rosmarinic acid	111.24 ± 13.39	40.16 ± 4.84	19.13 ± 1.99		6.94 ± 0.72	48.89 ± 3.34	18.20 ± 1.25	49.27 ± 8.55	18.92 ± 3.28
		**Flavan-3-ol**									
29–30	280	(−)epicatechin	324.44 ± 25.72	117.18 ± 9.288	228.36 ± 80.13		64.13 ± 38.46	431.99 ± 54.76	160.81 ± 20.18	222.03 ± 4.83	85.26 ± 1.86
		**Oligomer of flavan-3-ol**									
33–34	280	Procyanidin C1	247.35 ± 97.76	89.33 ± 35.31	64.45 ± 20.90		17.46 ± 11.60	224.85 ± 29.67	83.70 ± 11.05	75.14 ± 3.75	28.85 ± 1.44
		**Quercetin and glycosides**									
46–48	360	Quercetin-3-O-rutinoside (rutin)	3,366.6 ± 424.81	1,215.90 ± 153.43	1,317.19 ± 69.50		478.12 ± 25.23	636.52 ± 93.09	236.95 ± 34.28	544.24 ± 36.20	208.99 ± 13.90
50–52	360	Quercetin-3-O-glucoside (isoquercetin)	1,331.74 ± 133.24	480.98 ± 48.11	303.18 ± 5.42		130.08 ± 34.72	302.34 ± 43.76	112.54 ± 16.29	260.86 ± 13.70	100.17 ± 5.26
52–54	360	Quercetin-3-rhamnoside (quercitrin)	2,714.28 ± 231.37	980.31 ± 83.56	1,000.79 ± 71.36		363.29 ± 25.91	629.23 ± 97.81	234.23 ± 36.41	534.22 ± 26.20	205.14 ± 10.06
67–68	360	Quercetin	87.185 ± 6.09	31.49 ± 2.20	142.40 ± 18.67		51.69 ± 6.78	4.76 ± 0.30	1.77 ± 0.11	14.89 ± 0.83	5.72 ± 0.32
		**Other flavonoids**									
54–56	360	Myricetin	47.54 ± 0.98	17.17 ± 0.35	36.25 ± 6.18		13.16 ± 2.25	47.60 ± 4.77	17.72 ± 1.78	26.99 ± 3.85	10.36 ± 1.48
70–72	360	Kaempferol	3.98 ± 1.59	1.44 ± 0.57	9.64 ± 0.34		2.49 ± 1.75	**–**	**–**	**–**	**–**
58–59	280	Phloridzin	456.94 ± 24.21	165.03 ± 8.74	962.82 ± 2.74		361.27 ± 20.39	188.99 ± 25.46	70.35 ± 9.48	163.94 ± 7.98	62.95 ± 3.06
		Total phenolics (TPTC)	10,195.56 ± 567.28	3,682.30 ± 204.88	5,559.67 ± 365.50		2,024.10 ± 132.68	3,933.64 ± 504.70	1,464.30 ± 187.87	3,000.58 ± 93.74	1,152.94 ± 36.00

**Table 3:** The HPLC analysis of triterpenoids and organic acids (including ascorbic acid) in the of Papierowka and Gold Milenium apple peels [mg/g d.w. extract or peel].

	Papierowka				Gold Milenium			
	Ultrasound		Conventional		Ultrasound		Conventional	
	mg/g d.w. extract	mg/g d.w. peel	mg/g d.w. extract	mg/g d.w. peel	mg/g d.w. extract	mg/g d.w. peel	mg/g d.w. extract	mg/g d.w. peel
**Triterpenoids**								
Betulinic acid	0.145 ± 0.032*	0.053 ± 0.015	0.180 ± 0.0159	0.059 ± 0.009	0.058 ± 0.003	0.021 ± 0.001	0.040 ± 0.005	0.013 ± 0.001
Oleanolic acid	0.085 ± 0.007	0.020 ± 0.001	0.097 ± 0.010	0.032 ± 0.006	0.156 ± 0.002	0.039 ± 0.006	0.194 ± 0.019	0.064 ± 0.007
Ursolic acid	3.507 ± 0.102	1.351 ± 0.083	3.373 ± 0.027	1.107 ± 0.113	1.638 ± 0.006	0.324 ± 0.022	2.090 ± 0.018	0.688 ± 0.063
**Organic acids**								
Tertaric acid	3.748 ± 0.034	1.353 ± 0.172	5.283 ± 0.074	1.741 ± 0.153	0.0710 ± 0.054	0.256 ± 0.020	0.100 ± 0.0161	0.033 ± 0.005
Malic acid	55.709 ± 0.118	20.086 ± 2.383	66.523 ± 0.718	21.929 ± 2.150	24.318 ± 0.819	8.783 ± 0.296	15.595 ± 0.031	5.138 ± 0.010
Ascorbic acid	0.016 ± 0.000	0.006 ± 0.001	0.000	0.000	0.014 ± 0.001	0.005 ± 0.000	0.000	0.000
Citric acid	1.006 ± 0.135	0.408 ± 0.099	1.540 ± 0.115	0.457 ± 0.041	0.278 ± 0.009	0.101 ± 0.003	0.253 ± 0.143	0.083 ± 0.047
Quinic acid	48.007 ± 0.076	17.310 ± 2.068	67.626 ± 0.645	22.306 ± 2.405	10.412 ± 0.100	3.760 ± 0.036	1.926 ± 0.027	0.635 ± 0.009

*Mean values from three independent experiments ± SD are shown.

## Anti-/pro-oxidant activity of apple peels

### DPPH, ABTS, FRAP and CUPRAC assays

In both DPPH and ABTS tests Papierowka peels (i.e. DPPH: 0.097 ± 0.015 mg/mL; ABTS: 0.061 ± 0.009 mg/mL) had higher antioxidant activity than Gold Milenium peels (e.g. DPPH: 0.142 ± 0.011 mg/mL; ABTS: 0.095 ± 0.003 mg/mL) (Table 4). The differences between the activity of extract obtained by ultrasound assisted and conventional extraction of Papierowka peels were slight. Whereas more distinct differences were shown in the case of Gold Milenium peel extracts obtained by these two types of extraction. In the DPPH assay the conventional extraction was more efficient method of antioxidant extraction from Gold Milenium peel. On the other hand, according to ABTS assay the extract obtained by ultrasound assisted extraction was a better radical scavenger than the extract obtained by conventional extraction. Similar dependency was determined in the case of Papierowka peel extracts, but the differences between these types of extraction were not so evident. Generally, the content of phenolic compounds in the peel extracts determined in the method with F–C reagent correlates with their ability to scavenge the ABTS^+^· radical cation.

**Table 4:** The antioxidant activity (in DPPH, ABTA, FRAP, CUPRAC assays) of apple peels obtained by conventional and ultrasound assisted extraction.

Apple variety	Ultrasound assisted	Conventional	Ultrasound assisted	Conventional
	DPPH/EC_50_ [mg/mL]		ABTS/EC_50_ [mg/mL]	
Gold Milenium	0.142 ± 0.011*	0.119 ± 0.004	0.095 ± 0.003	0.115 ± 0.020
Papierowka	0.097 ± 0.015	0.095 ± 0.011	0.061 ± 0.009	0.070 ± 0.014

	FRAP [μM_Fe2+_]			
	0.39 mg/mL**		0.78 mg/mL	
Gold Milenium	57.182 ± 6.214**	58.647 ± 0.273	118.122 ± 6.428	125.960 ± 3.436
Papierówka	95.990 ± 1.881	89.715 ± 0.714	197.327 ± 5.415	110.557 ± 14.474

	CUPRAC/Trolox equivalents [μM]			
	0.39 mg/mL		0.78 mg/mL	
Gold Milenium	76.720 ± 6.437	91.160 ± 9.459	145.560 ± 8.406	150.733 ± 14.088
Papierówka	102.513 ± 6.534	97.813 ± 2.282	227.210 ± 10.102	212.127 ± 7.563

*Mean values from three independent experiments ± SD are shown.

**0.39 and 0.78 mg/mL—the tested concentration of ethanolic extract of apple peels.

The FRAP and CUPRAC values showed that the extracts had a ferric and cupric reducing antioxidant power and with the increase in the concentration of extracts their antioxidant activity increased as well (Table 4). Papierowka peels possessed higher reducing activity than Gold Milenium peels (except of conventional extraction of Gold Milenium, C = 0.78 mg/mL). In the case of Papierowka peel extract a distinct relationship between the type of extraction and the reducing activity was found. According to FRAP and CUPRAC assays the ultrasound assisted extraction is favourable for extracting the antioxidant compounds from Papierowka peels.

### Lipid peroxidation assay

**Figure 3:**
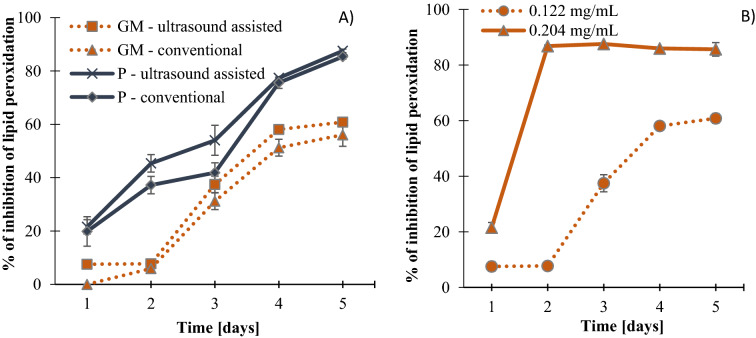
% of inhibition of lipid peroxidation by Gold Milenium (GM) and Papierowka (P) peel extracts at the concentration 0.122 and 0.204 mg/mL measured within 5 days. Mean values from three independent experiments ± SD are shown.

Both Gold Milenium and Papierowka peel extracts inhibited the peroxidation of lipids (Fig. 3) which increased within the five days of measurement. Extracts obtained by ultrasound assisted extraction inhibited the lipid peroxidation to a greater extend. Moreover Papierowka peel extracts inhibited the lipid peroxidation about 10–30% better than extract of Gold Milenium peels. With the increase in the concentration of extract the antioxidant activity measured as inhibition of lipid peroxidation increased as well (Fig. 3). In the case of the concentration 0.204 mg/mL the inhibition of lipid peroxidation reached the maximum value in the second day of measurement and it is at the constant level within the next three days.

### Pro-oxidant assay

**Figure 4:**
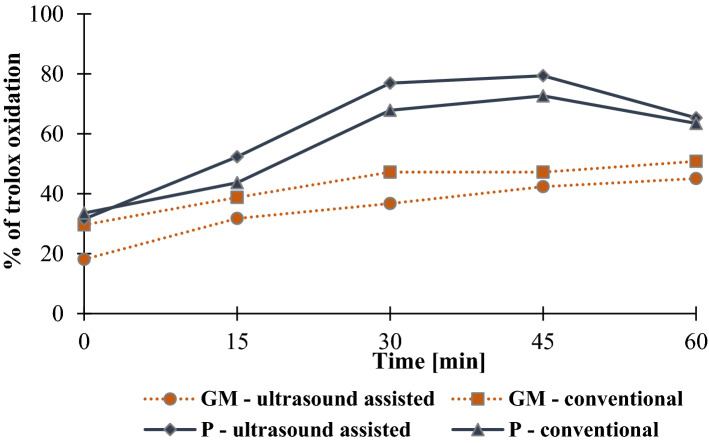
The degree of trolox oxidation [%] in the presence of Gold Milenium (GM) and Papierowka (P) peel extracts (at the concentration 3.75 μg/mL) obtained by ultrasound assisted and conventional extraction.

**Figure 5:**
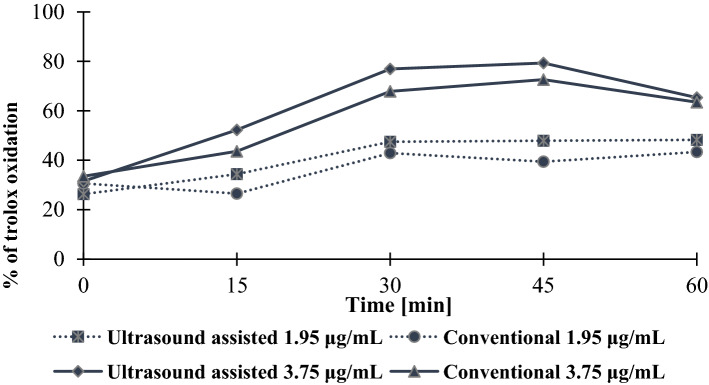
The degree of trolox oxidation [%] in the presence of Papierowka peel extracts (at the concentrations 1.95 and 3.75 μg/mL) obtained by ultrasound assisted and conventional extraction.

The extract of Papierowka peel (more abundant in phenolic compounds compared to Gold Milenium peel extract) showed higher pro-oxidant activity (Figs. 4, 5). Moreover, extracts obtained by ultrasound assisted extraction caused oxidation of trolox to a greater extend compared to conventionally obtained extracts.

### Cytotoxicity study (MTT test)

**Figure 6:**
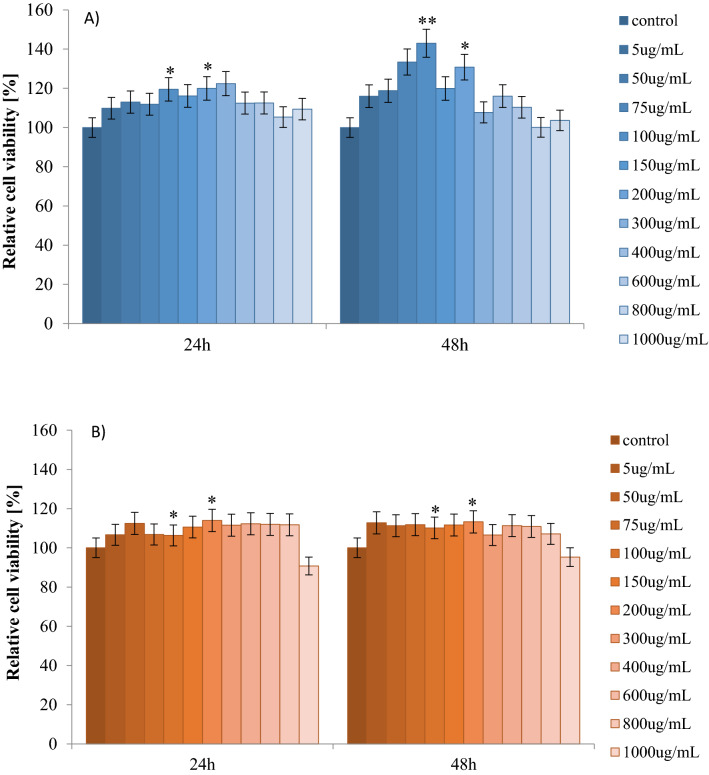
Cell viability results using MTT assay for fibroblast cells exposed to graded concentrations of (**A**) Papierowka and (**B**) Gold Milenium for 24 h and 48 h calculated as a percentage of control, untreated cells. Each value on the graph is the mean of three independent experiments and error bars show the standard error of means (SEM). *p < 0.05 and **p < 0.01 represent significant effects between treatments and control followed by a Dunnett’s test.

The cytotoxicity of apple peel extracts at various concentrations (5–1,000 μg/mL) on fibroblast cells was measured in MTT assay. In this and the following studies (determination of SH groups and TBARS level) only the peel extracts obtained by ultrasounds extraction were tested, because they had higher phenolic content and antioxidant activity compared to conventionally obtained extracts. Therefore, we assumed to observe higher biological effect on the selected oxidative stress parameters in fibroblasts. Results of MTT assays are presented in Fig. 6. On the basis of the obtained in MTT assay results the 100 and 200 μg/mL concentrations of plant extract were selected for the study of oxidative stress/ TBARS content and thiol group level.

### Determination of SH groups

**Figure 7:**
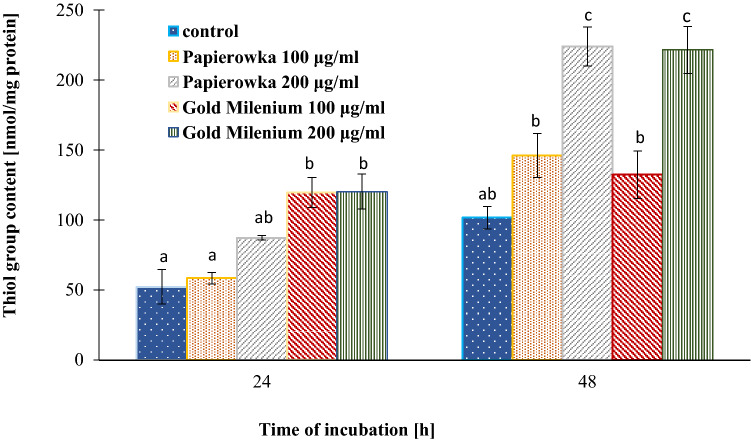
The effect of Papierowka and Gold Milenium apple peel extracts obtained by ultrasound assisted extraction on SH group content in fibroblast cells. Mean values from three independent experiments ± SD are shown.

The thiol group content was evaluated as a marker of protein oxidation. The effect of apple peel extracts on SH group content was shown in Fig. 7. The exposure to Papierowka peel extract resulted in ~ 12% (C = 100 μg/mL) and ~ 67% (C = 200 μg/mL) increase in the total cellular content of the thiol groups after 24 h treatment. The stronger effect was observed in the case of Gold Milenium extract and reached ~ 130% (C = 100 and 200 μg/mL) increase in the content of the thiol groups. After 48 h of treatment the extract still caused the increase in the contend of the thiol group to the ~ 35% (C = 100 μg/mL) and ~ 118% (C = 200 μg/mL) compared to the control sample. The data indicated that Papierowka and Gold Milenium peel extracts did not cause oxidative damage in proteins. This effect increased with the increase in the concentration of extracts.

### Determination of TBA reactive species (TBARS) levels

**Figure 8:**
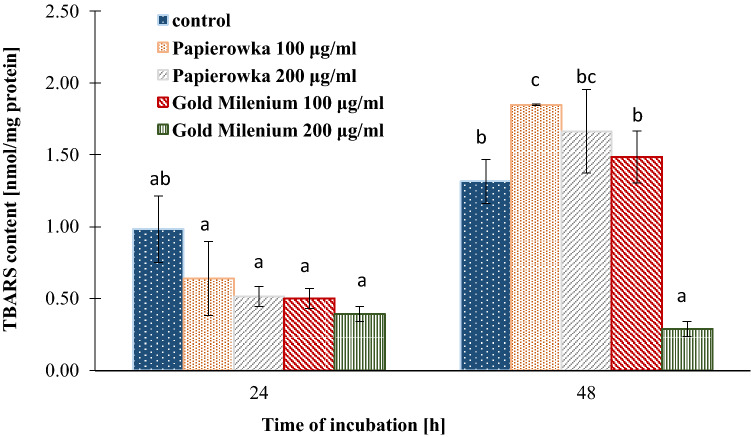
The effect of Papierowka and Gold Milenium apple peel extracts obtained by ultrasound assisted extraction on TBARS content in fibroblast cells. Mean values from three independent experiments ± SD are shown.

Lipid peroxidation is a process connected with a variety of cellular dysfunctions, which results from the inappropriate modifications of lipid-protein complexes. TBARS content was measured as an index of lipid peroxidation. The results showed that the Papierowka and Gold Milenium apple peel extracts at the concentrations of 100 and 200 µg/mL induced a decrease in TBARS content to the level of ~ 35–60% compared to the control observed after 24 h of incubation (Fig. 8). After 48 h of treatment the Papierowka peel extract caused increase in the TBARS content to ~ 40% (C = 100 μg/mL) and ~ 16% (C = 200 μg/mL). The extract of Gold Milenium peels at the concentration 100 μg/mL showed lower raise in the TBARS content to 13%. Whereas the Gold Milenium peel extract at the concentration 200 μg/mL caused significant reduction in TBARS content.

## Discussion

Peel possess about 5 times more phenolics than flesh (5.7 times more in the case of Papierowka and 4.1 times more for Gold Milenium variety) and twice as much as core (Papierowka: 2.5 times more; Gold Milenium: 1.8 times more). This is in accordance with other studies, e.g. according to Kschonsek et al. flesh had a 3.0–28.4 times lower content of polyphenols than peel^15^; Łata et al. showed that peel expressed 2–9 times higher content of phenolics than the pulp^31^. Although, the total content of phenolic compounds is higher in apple peel than flesh, some individual compounds may be more abundant in flesh than peel depending on the apple variety. E.g. more chlorogenic acid was found in the peel than the flesh of Fuji^32^, McIntosh^33^, Red Delicious^34^, Idared^35^, whereas Golden Delicious possessed more chlorogenic acid in flesh than peel^34^. p-Coumaroylquinic acid is more abundant in the flesh than peel of Gala variety^33^. Procyanidin B1 was found in higher amount in the flesh of Elstar^36^, Gala^33^, Gloster^36^. Generaly (+) and (−)-epicatechin, procyanidin B2, C1 and phloridzin were determined in higher amount in peel than flesh of apples (detailed data in^5^). The differences in the distribution of phenolic compounds in the apple tissues depends on their variety and can be explained in the terms of their biosynthesis pathway^37^. Fruit maturation as well as growth conditions (conventional or ecological farming, weather, soil type, light exposure, diseases), storage condition and processing affect the content of phenolics in fruits. The content of phenolic compounds is not stable and may change over years. Belviso et al. showed that during the three year period of study the content of chlorogenic acid and phloridzin differed significantly, whereas catechins and procyanidins were almost at the same level^38^.

Our studies revealed that extract of Papierowka peels possess much higher amount of phenolic compounds compared to Gold Milenium (Papierowka: 3.68 ± 0.20 mg/g peel u.a.e; 2.02 ± 0.13 mg/g peel c.e.; Gold Milenium: 1.46 ± 0.19 mg/g peel u.a.e; 1.15 ± 0.04 mg/g peel c.e. according the HPLC measurement). The results of the assay with the Folin–Ciocalteau reagent also revealed much higher content of antioxidants in the Papierowka peels compared to Gold Milenium variety. On the other hand the HPLC analysis showed much difference in the phenolic compound composition comparing the ultrasound assisted and conventional extractions, whereas the assay the F–C reagent pointed at almost lack of the diversity of composition taking into account these two types of extraction. Maybe phenolic compounds are one of the many different groups of chemicals that react with the F–C reagent and they have a minor contribution to the final value. Those chemicals are extracted from the apples in similar quantities irrespective to the type of extraction because are not so tightly bound to the cell wall in plants (e.g. simple organic acids, reducing sugars, ascorbic acid or minerals). The data from Table 1 indicated that the peels of Papierowka are more abundant in reducing sugars as well as iron and copper than Gold Milenium. Those chemicals are reducing interferants that react with the F–C reagent and enhance the determined value. A few attempts were done to improve the methodology with F–C reagent applied to study the content of phenolic compounds^39,40^.

Chlorogenic acid, homovanilic acid, (−)-epicatechin, rutin, quercetin-3-glucoside, quercetin-3-rhamnoside and phloridzin are the main phenolic compounds found in the peels of both apple varieties (Papierowka: 0.177–1.216 mg/g d.w.; Gold Milenium: 0.070–0.237 mg/g d.w.—taking into account the ultrasound assisted extraction). Moreover, Gold Milenium peels are abundant in ellagic acid which content is about six times higher than in Papierowka peels. The other phenolic compounds, i.e. myricetin, quercetin, kaempferol, gallic, protocatechuic, vanilic and rosmarinic acid were also detected. These two varieties differ mainly in the content of rutin, quercetin-3-glucoside, quercetin-3-rhamnoside, quercetin, vanilic and protocatechuic acis which are from 4 to 18 more abundant in Papierowka than Gold Milenium.

Both varieties possess moderate content of chlorogenic acid (Papierowka: 0.338 mg/g d.w.—conventional extraction; Gold Milenium: 0.176 mg/g d.w.—ultrasound extraction). Huber and Rupasinghe evaluated the phenolic compound composition of peels of 21 apple varieties^41^. The average content of chlorogenic acid was 0.42 ± 0.32 mg/g d.w. According to Kschonsek et al. the amount of chlorogenic acid in the peels of 15 different apple varieties ranged from 0.039 ± 0.016 to 0.18 ± 0.065 mg/g freeze dried material (f.d.m.)^15^. Łata et al. evaluated much higher content of chlorogenic acid in the peels of 19 varieties of apple, i.e. 2.33–0.26 mg/g d.w.^31^. Papierowka and Gold Milenium peels possess high content of rutin, i.e. 1.216 and 0.237 mg/g d.w (ultrasound assisted extraction), respectively. The literature data reported the concentration of rutin in apple peels in the range of 0.09–0.24 mg/g f.d.m.^15^, 0.7–1.1 mg/g d.w.^40^ or even 1.24–5.75 mg/g d.w.^31^. Quercetin-3-rhamnoside content was 0.980 ± 0.084 mg/g d.w. (Papierowka) and 0.234 ± 0.036 mg/g d.w. (Gold Milenium). This is relatively high quantity comparing the data obtained by Huber and Rupasinghe (0.59 ± 0.42 mg/g d.w.)^42^ and Kschonsek et al. (0.15 ± 0.43–0.84 ± 0.61 mg/g f.d.m.)^15^ and others 0.12–2.66 mg/g d.w.^43–45^. Quercetin-3-glucoside was evaluated at the following level: 0.480 ± 0.048 mg/g d.w. (Papierowka) and 0.113 ± 0.016 mg/g d.w. (Gold Milenium) whereas others reported 0.06 ± 0.04–0.45 ± 0.28 mg/g f.d.m.^15^, 0.23 ± 0.18 mg/g d.w. (Huber and Rupasinghe, 2009)^42^, 0.53–4.00 mg/g d.w.^43^. Procyanidin C1 was determined at the concentration of 0.089 ± 0.035 mg/g d.w. (Papierowka) and 0.084 ± 0.011 mg/g d.w. (Gold Milenium). This is similar to Kolodziejczyk and Kosmala^36^—0.097–0.213 mg/g d.w. and much higher than reported Kschonsek et al. ~ 0.014 mg/g f.d.w.^15^. Phloridzin concentration in apple peel reached 0.361 ± 0.020 mg/g d.w. (Papierowka) and 0.070 ± 0.009 mg/g d.w. (Gold Milenium). It is much lower than determined by Łata et al.—average value 1.55 mg/g d.w.^31^ and similar to the data reported by Kschonsek et al. ~ 3.79 mg/g f.d.w.^15^ depending on apple variety.

The ultrasound assisted extraction enhance the yield of extraction of phenolics to 83% and 31% in the case of Papierowka and Gold Milenium peels, respectively. It concerns quercetin glucosides, flavan-3-ols, some of the phenolic acid (vanilic, homovanilic, ellagic, rosmarinic), myricetin which extraction efficiency increased several times after ultrasounds application. On the other hand there were other simple phenolic acids (gallic, protocatechuic, chlorogenic) and quercetin, phloridzin, kaempferol which yield of extraction decreased when ultrasounds were used. This may be caused by the degradation and/or oxidation of these phenolic compounds due to the production of free radicals during the process of ultrasound assisted extraction^19^. Therefore the extraction should be optimized depending on the type of phenolic compounds.

Because the antioxidant activity of plant extract directly depends on the phenolic compounds content it was assumed that that extracts abundant in phenolic compounds would possess higher antioxidant property in applied assays^5^. As it was supposed, the extract of Papierowka peel revealed higher antiradical (in DPPH and ABTS tests), reducing (in FRAP and CUPRAC assays) and lipid peroxidation inhibitory activities compared to the Gold Milenium peels. The extracts of both varieties obtained by ultrasound assisted extraction showed higher content of phenolic compounds, so it was expected that these extracts had higher antioxidant activity compared with the extracts obtained by conventional extraction. It revealed that the results of DPPH did not correspond with the total content of phenolic compounds found in the extracts obtained by two different methods of extraction. The lower values of EC_50_ (DPPH) were determined for the extracts from the two varieties extracted conventionally. It means that these extracts showed better antiradical activity than these ones obtained by the use of ultrasounds. The inverse relationship was achieved in the case of ABTS assays—the lower EC_50_ parameters were determined for the extracts obtained by ultrasound assisted extraction than conventional, what is in accordance with the total content of phenolic compounds. The negative or poor correlation of the DPPH results with the content of phenolic compounds in plant extract was reported previously by other authors^46–48^. The antioxidant properties of extracts examined by the use of DPPH methods are mainly affected by the type of solvent, metal ions and pH of the solution^19,49,50^. DPPH assay is sensitive to acidic pH—the increase in the concentration of hydrogen ions cause a decrease in the antioxidant activity of tested sample^51^. It is assumed that an increase in the acidity leads to decrease of the antioxidant/DPPH· reaction rate^50^. The same effect may occur in the case of the presence of metal cations in the reactive medium which may block the scavenging reaction of DPPH·. Because plant extracts are abundant in phenolic compounds (and the concentration of phenolic compounds is a decisive parameter of the antioxidant activity of extract) the interactions of phenolic-metal ion may influence the antioxidant activity of sample. Some papers postulated that the presence of metal ions may increase the antioxidant activity of phenolic compounds because metal coordination change the redox potential of ligand and increase in the electron-donating ability of phenolics^52–54^. However, there are other studies which revealed that the antioxidant properties of phenolic compounds decreased upon complexation^51,53,55^ or the oxidant activity changed from anti- to pro-oxidant when the lifetime of phenoxyl radicals is prolonged due to spin-stabilizing effect induced by metal cations^56^.

Probably due to above mentioned factors that may affect the results obtained by the DPPH many authors indicated better correlation between total phenolic, ABTS and FRAP values^57,58^. The obtained FRAP and CUPRAC values pointed at better antioxidant reducing properties of Papierowka than Gold Milenium peel extracts. With the increase in the concentration of extracts (from 0.38 to 0.79 mg/mL) the reducing activity and inhibition of lipid peroxidation increased. As it was supposed, the extracts from the Papierowka peel better inhibited the lipid peroxidation compared to Gold Milenium due to higher total phenolic content. The extracts obtained by ultrasound assisted extraction revealed higher antioxidant activity measured as the inhibition of lipid peroxidation than the conventionally prepared extracts.

**Figure 9:**
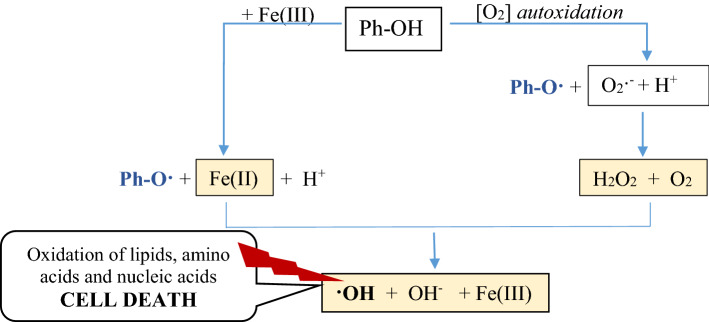
The possible way of phenoxyl radicals and reactive oxygen species production. The yellow field indicate the Fenton reaction. Ph-OH means phenolic compound. The phenolic compound may undergo autoxidation to produce phenoxyl radical, superoxide radical anion (O_2_·^−^) and hydrogen cation (H^+^). O_2_·^−^ and H^+^ react to form hydrogen peroxide and oxygen. In the presence of same transition metals (e.g. Fe(III)) the phenoxyl radicals and reduced metal cations (Fe(II)) are produced. Finally, H_2_O_2_ is reduced to ·OH and Fe(II) is oxidised to Fe(III) in the Fenton reaction.

Pro-oxidant activity of extract was studied as a measure of the oxidation of trolox by phenoxyl radicals produced in the reaction of phenolic compounds with H_2_O_2_ catalysed by horse radish peroxide. The extract with the highest content of phenolic compounds, i.e. Papierowka peel extract obtained by ultrasound assisted extraction, showed the highest pro-oxidant activity. The pro-oxidant activity of extracts increased with their concentration. In this assay the pro-oxidant activity is measured as the ability to form phenoxyl radicals. It should be kept in mind that the pro-oxidant activity of plant extract is generally related to the production of reactive oxygen species ROS (·OH, O_2_·^−^, H_2_O_2_, ROO·) which may be initiated by phenolic compounds. The additional presence of transition metals such as Cu(II) or Fe(III) enhance the production of ROS (Fig. 9). Moreover the pro-oxidant activity of plant extracts may increase when the lifetime of phenoxyl radicals is enhance due to their stabilization by metal cations^56^.

Phenoxyl radical, superoxide radical anion (O_2_·^−^) and hydrogen cation (H^+^). O_2_·^−^ and H^+^ react to form hydrogen peroxide and oxygen. In the presence of same transition metals (e.g. Fe(III)) the phenoxyl radicals and reduced metal cations (Fe(II)) are produced. Finally, H_2_O_2_ is reduced to ·OH and Fe(II) is oxidised to Fe(III) in the Fenton reaction.

The Papierowka and Gold Milenium peel extracts obtained by ultrasound assisted extraction were studied for their influence on basic oxidative stress parameters in cultured human skin fibroblasts, i.e. the SH groups and TBARS were established. The exposure to peel extract at the concentration 100 and 200 μg/mL resulted in the increase in the total cellular content of the thiol groups. This effect increased with the increase in the concentration of extracts and lasted up to 48 h later. Moreover, after 24 h of incubation the extracts caused a decrease in TBARS content at both tested concentrations. Whereas, after 48 h of incubation only Gold Milenium extract at the concentration 200 μg/mL still showed protective properties against TBARS production and decreased membrane phospholipid peroxidation. The results point that apple peel extract is promising agent reducing the oxidative stress in skin fibroblast. The elimination of the oxidative stress in skin fibroblast by plant extracts is usually related with their antioxidant properties and the content of phenolic compounds^59–61^. Facial serum containing apple stem cell extract reduces the mitochondrial ROS production in human senescent cultured fibroblasts^62^. Many of the phenolics present in apple peel extract were tested for their protective activity against oxidative stress in human skin fibroblast. Rutin, the main phenolic compounds present in apple peel, prevented against the increased in phospholipase A2 activity and ROS generation in UV-induced skin fibroblasts^63^. The level of arachidonic and linoleic acids level was not reduced and the content of 4-hydroxynonenal (a product of lipid peroxidation) did not increased. Isoquercitrin, abundant in apple peel extract, inhibited UVB irradiation-induced oxidative damage in the JB6 P^+^ mouse epidermal C141 cells. The results showed that quercitrin restored catalase expression and GSH/GSSG ratio^64^. Quercetin protected human skin fibroblasts against oxidative stress induced by buthionine sulfoximine which is an inhibitor of glutathione^65^. Our previous studies showed that cichoric acid (strong plant antioxidant) exhibits cytoprotective activity against DOX-induced damage in skin fibroblasts by lowering oxidative stress level and by inhibiting apoptosis^66^.

## Conclusions

The variety of apple and the type of extraction significantly affect the composition of apple peel extracts. The commonly applied antioxidant tests may be affected by the complex matrix of plant extracts. Generally, the peels of Papierowka possess significantly higher amount of phenolics triterpenoids, simple organic acid and are more abundant in selected macro-, microelements and reducing sugar than Gold Milenium peels. Moreover, the ultrasound assisted extraction is more efficient in phenolic compounds extraction from apple peels compared with the conventional extraction, although some of the individual phenolics are better extractable by the conventional technique. Plant extracts of both varieties are rich in phenolic compounds and can applied widely in environmental pollution control, agriculture production, food industry or medicine.

## Supplementary information

Supplementary information.
