# Supplementary material for: Phenolic content, chemical composition and anti-/pro-oxidant activity of Gold Milenium and Papierowka apple peel extracts
Source: Sci Rep. 2020 Sep 11;10:14951. doi: 10.1038/s41598-020-71351-w (PMC7486935; doi:10.1038/s41598-020-71351-w)
Supplement: Supplementary file 1 — Supplementary information. [file 41598_2020_71351_MOESM1_ESM.pdf]

Supplementary data

Chemical composition, anti-/pro-oxidant activity of apple peel extracts - environmental friendly source of bioactive compounds with possible application in agricultural, food and pharmaceutical industries

Monika Kalinowska[a*], Kamila Gryko[a], Anna M. Wróblewska[b], [A]gata Jabłońska-Trypuć[a], Danuta Karpowicz[a]

[a] Department of Chemistry, Biology and Biotechnology, Bialystok University of Technology, Wiejska 45A, 15-351 Bialystok, Poland

[b] Faculty of Chemistry, Warsaw University of Technology, Noakowskiego 3, 00-664 Warsaw, Poland

[*] CONTACT: Monika Kalinowska, e-mail: m.kalinowska@pb.edu.pl; address: Department of Chemistry, Biology and Biotechnology, Bialystok University of Technology, Bialystok, Poland

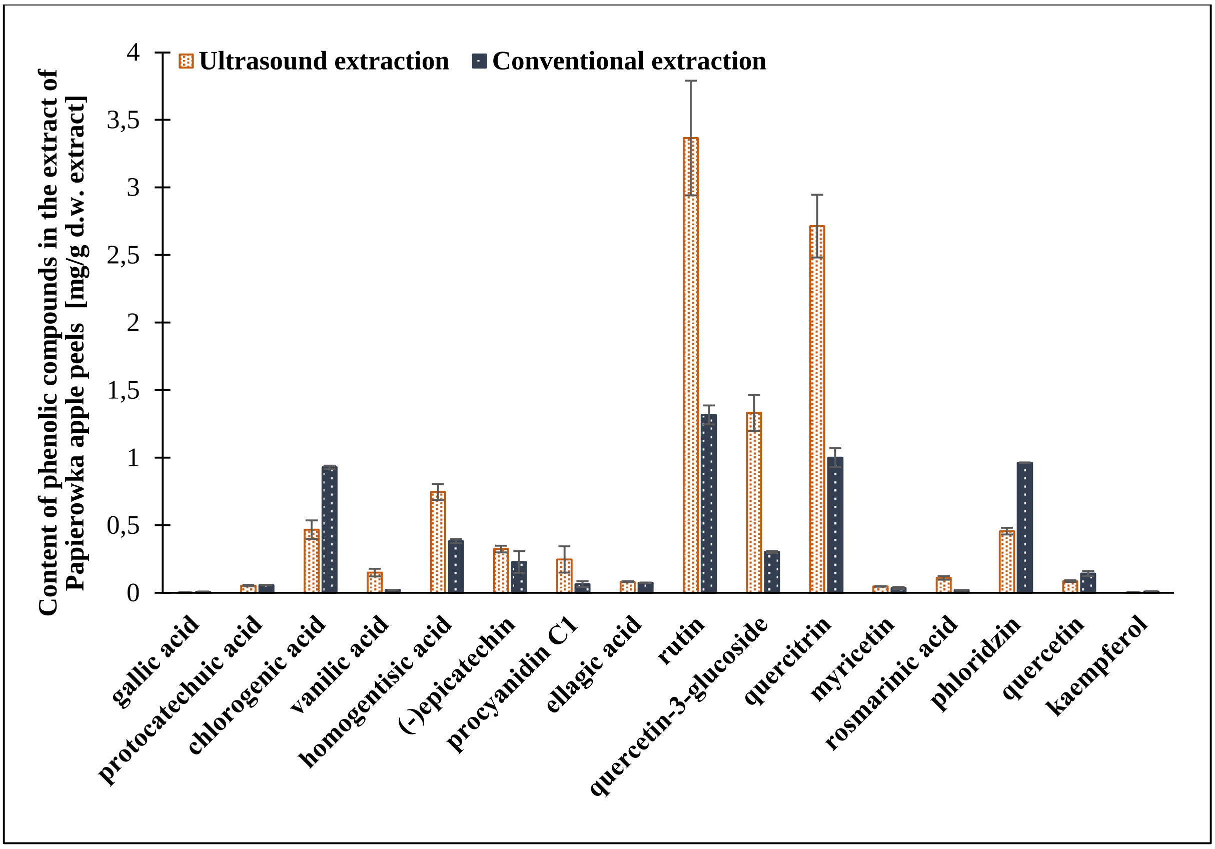

Figure S1. The content of particular phenolic compounds in Papierowka apple peel extract. Mean values from three independent experiments ± SD are shown.

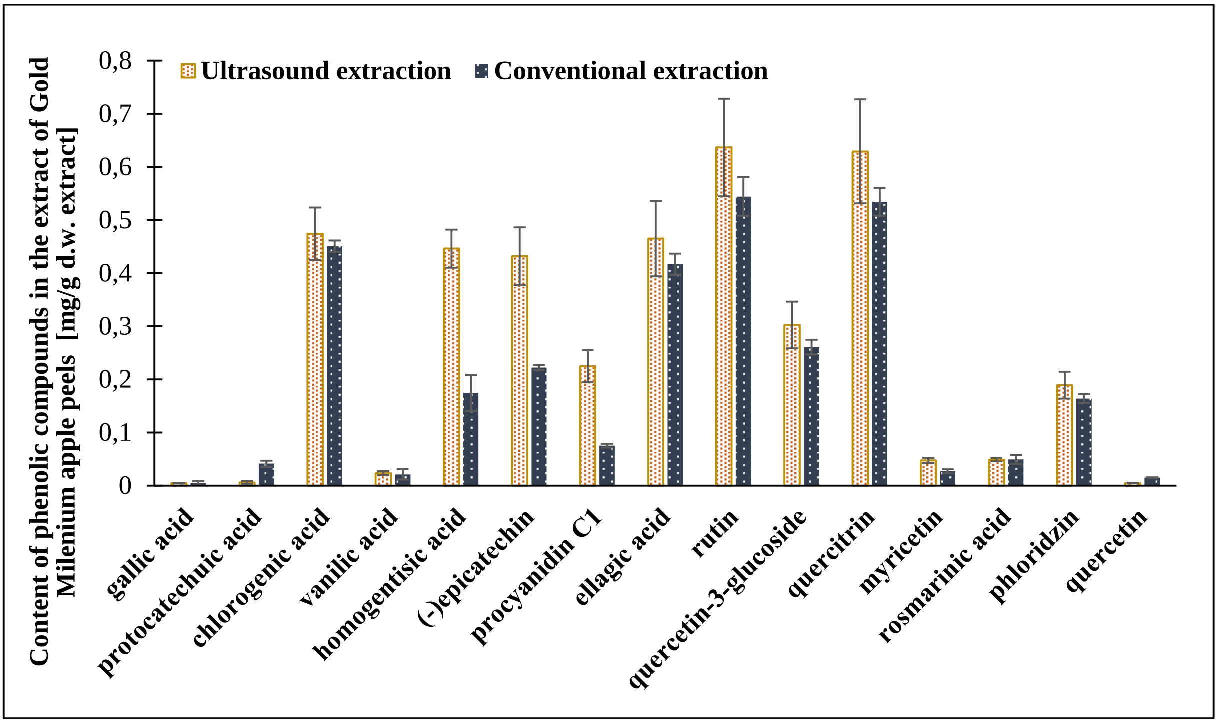

Figure S2. The content of particular phenolic compounds in Gold Milenium apple peel extract. Mean values from three independent experiments ± SD are shown.
